# T_1_ Shortening in the Cerebral Cortex after Multiple Administrations of Gadolinium-based Contrast Agents

**DOI:** 10.2463/mrms.mp.2016-0054

**Published:** 2016-10-11

**Authors:** Zaw Aung Khant, Toshinori Hirai, Yoshihito Kadota, Rie Masuda, Takanori Yano, Minako Azuma, Yukiko Suzuki, Kuniyuki Tashiro

**Affiliations:** 1 Department of Radiology, Faculty of Medicine, University of Miyazaki, Miyazaki, Japan; 2 Department of Radiology, Junwakai Memorial Hospital, Miyazaki, Japan; 3 Department of Radiology, Fukuoka Wajiro Hospital, Fukuoka, Japan

**Keywords:** T_1_ shortening, cerebral cortex, gadolinium

## Abstract

We report a 34-year-old male who manifested T_1_ shortening of the cerebral cortices after more than 86 contrast-enhanced MRI studies. We observed high-signal intensity (SI) on T_1_-weighted images (T_1_WIs) not only in the globus pallidus, dentate nucleus, and pulvinar of thalamus, but also in the cortices of the pre- and post-central gyri and around the calcarine sulcus. High SI in the cerebral cortices was not clearly demonstrated on T_1_WI scans performed 11 years earlier. The high SI we observed in these areas of the brain corresponded to areas with a normal iron-deposition predilection. Gadolinium deposition in the brain may be associated with the iron metabolism.

## Introduction

Gadolinium (Gd) deposition in the brain has been reported after the iterative administration of Gd-based contrast agents (GBCAs) even in patients with normal renal function; the globus pallidus and dentate nucleus are GBCA-related hyperintensity areas on T_1_WIs.^1–5^ The signal intensity (SI) and residual Gd in these brain regions reflect the total dose of GBCAs administered over time.^1–3,6,7^ As free Gd is cytotoxic, it is delivered in chelated form.^8^ High SI on T_1_WIs varies with the chelate type of previously administered GBCAs and is correlated with the administration of linear but not macrocyclic chelate GBCAs because linear-type is more unstable than macrocyclic-type GBCAs.^4,9–11^ High SI in the cerebral cortices on T_1_WIs of patients with previous administrations of GBCAs has not been reported. We document T_1_ shortening of the cerebral cortices in a patient who had undergone multiple GBCA-enhanced studies.

## Case Report

This 34-year-old man presented with bilateral hearing loss at the age of 14. He harbored bilateral acoustic and spinal schwannomas and was diagnosed with neurofibromatosis type 2. At the age of 33 he was also diagnosed with meningioma and he underwent many surgical and radiotherapy (e.g., r-knife, cyber-knife) treatments. For pre- and post-treatment evaluations he had undergone multiple contrast-enhanced MR studies using GBCAs. He manifested no renal dysfunction.

We suspect that contrast-enhanced MR examinations had been performed before 2003, but we were not able to obtain the information about the administration of GBCAs before 2003 because of no records remained for that. We confirmed that between 2003 and 2014, 86 GBCA-enhanced studies had been performed; 59 were gadopentetate dimeglumine (Magnevist; Bayer Yakuhin, Osaka, Japan)-, 24 gadoterate meglumine (Magnescope; Terumo, Tokyo, Japan)-, and 3 gadoteridol (ProHance; Eisai, Tokyo, Japan)-enhanced. Most often the linear-type ionic contrast agent gadopentetate dimeglumine had been administered. MRI scans acquired in 2003 were the earliest images available to us. In 2003, the studies were performed on a Philips (Marconi) MAGNEX Eclipse 1.5T scanner (T_1_WI, TR/TE = 500 ms /11.4 ms; slice thickness = 5 mm; matrix = 256 × 256; field-of-view (FOV) = 230 mm). Thereafter a GE SIGNA EXCITE Echo Speed Plus 1.5T scanner was used (T_1_WI, TR/TE = 450 ms /12 ms; slice thickness = 5 mm; matrix = 256 × 256; FOV = 230 mm). Although the scanners were different, the imaging parameters were similar.

**Fig 1.**
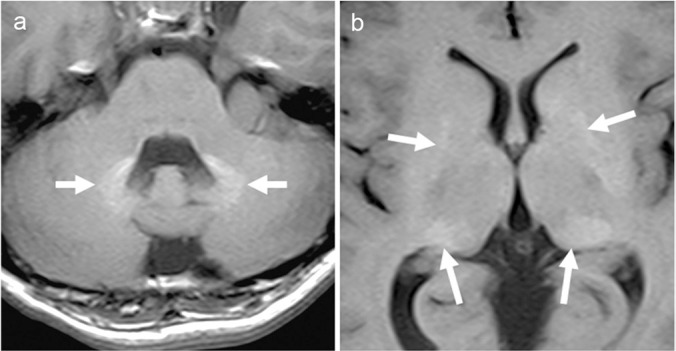
T_1_-weighted images obtained in 2003. (**a**) T_1_-weighted image shows high-signal intensity in the dentate nucleus (arrows) (**b**) T_1_-weighted image shows high-signal intensity in the globus pallidus and pulvinar of thalamus (arrows).

**Fig 2.**
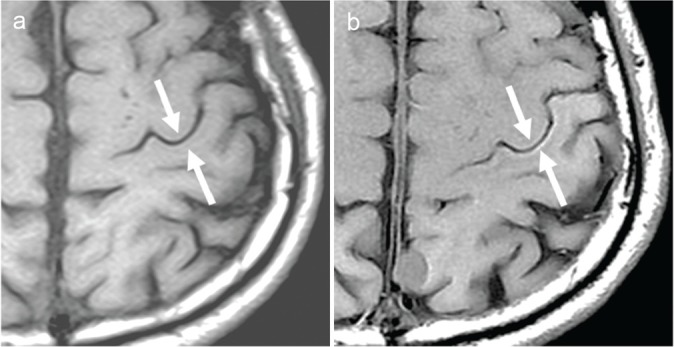
T_1_-weighted images at the level of the central sulcus. High-signal intensity can be seen more clearly on the 2014- (**b**) than the 2003 image (**a**) (arrows).

**Fig 3.**
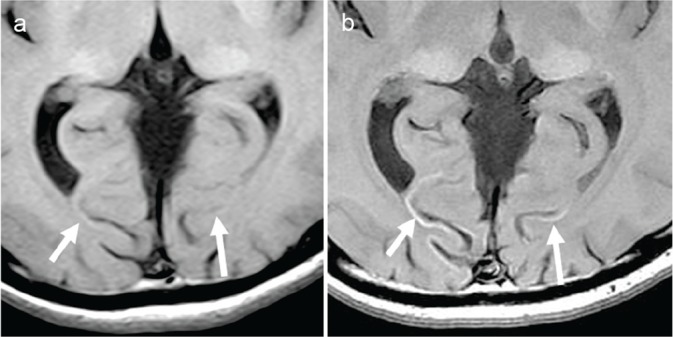
T_1_-weighted images at the level of the calcarine sulcus. High-signal intensity is more apparent on the 2014- (**b**) than the 2003 (**a**) image (arrows).

On the T_1_WIs acquired in 2003, we observed high SI in the globus pallidus, dentate nucleus, and pulvinar of thalamus (Fig. 1). On T_1_WI scans performed in 2014, we noticed prominent high SI not only in the globus pallidus, dentate nucleus, and pulvinar of thalamus, but also in the cortices of the pre- and post-central gyri and around the calcarine sulcus (Figs. 2 and 3).

## Discussion

High SI on T_1_WIs of the globus pallidus, dentate nucleus, and pulvinar of thalamus secondary to Gd deposition has been documented.^1–7^ However, our search of the literature found no reports of high SI attributable to Gd deposits in the cerebral cortices on T_1_WIs. Ours is the first documentation of high SI in the cortices of the pre- and post-central gyri and around the calcarine sulcus on T_1_WI scans of patients who had undergone multiple GBCA-enhanced imaging studies.

Although the mechanism(s) underlying Gd deposition in the brain is not fully understood, processes involved in the deposition of Gd in brain tissue have been suggested. Dechelation and transmetallation can give rise to a dissociation between Gd and its chelate.^12^ The possibility of the release of free Gd from the chelate in any GBCAs cannot be ruled out. Transmetallation, the exchange of Gd for other endogenous metal ions like iron, calcium, copper, and zinc that compete with Gd for chelation is also a possibility. The presence of free Gd in brain tissue suggests that Gd may be able to cross the blood brain barrier (BBB) even in the absence of evidence that the BBB has been compromised.^3^ As the passive transport of Gd is unlikely, some biological mechanism(s) such as metal transporter(s) may be involved.^7,13^

While iron circulating in the blood outside the central nervous system cannot cross the BBB directly, iron can be transferred across the BBB by several pathways. The probably most common is through transferrin receptors on brain endothelial cells; the receptors bind iron circulating in the form of transferrin. The transferrin receptor-bound complex then enters the brain by endocytosis. Other transporter systems such as the divalent metal transporter and the lactoferrin receptor may be involved in the delivery of iron across the BBB.^14,15^

The high SI in the deep gray matter and cerebral cortices on T_1_WI scans of our patient corresponded with areas identified in histochemical studies as areas with normal iron-deposition predilection.^16,17^ Spatz^16^ who reported a macroscopic, qualitative histochemical study of brain iron, divided the brain regions into four groups according to their iron content. The first, most intensely stained group was comprised of the globus pallidus and the substantia nigra. The second, consistently but less strongly stained group included the red nucleus, putamen, and the caudate- and dentate nucleus. The third group contained the cerebral and cerebellar cortex, the anterior thalamic nucleus, the mammillary body, and the tectum of the mid-brain; it was stained variably and considerably weaker. The regions in the fourth group showed no histochemical iron staining; they were the medulla oblongata, the gray matter of the spinal cord, and the white matter of the brain and spinal cord.

Hallgren and Sourander^17^ who studied the quantitative effect of age on non-heme iron in the human brain found that the iron content in the cerebral cortices increased with age; in the older study population the motor cortex (pre-central gyrus) had a mean iron content of approximately 5.0 mg/100 g, closely followed by the visual cortex (occipital cortex), the sensory cortex (post-central gyrus), and the rest of the parietal cortex. The temporal and pre-frontal cortices showed the lowest iron content (mean nearly 3.0 mg/100 g). The amount of iron deposition was highest in the motor-, followed by the visual- and the sensory cortex. They also detected nonheme iron in the globus pallidus, dentate nucleus, and thalamus; its content was higher in the globus pallidus (21–30 mg/100 g). These iron depositions in the deep gray matter and specific cerebral cortices were also observed on conventional spin-echo T_2_WI but not on fast spin-echo T_2_WI.^18–20^ On the other hand, no abnormal SI was observed in the motor and visual cortices on T_1_WI scans of healthy, even elderly individuals.^19,20^ We think that the T_1_ shortening of the cerebral cortices we observed in our patient was due to excessive Gd accumulation in the cortices after the multiple administrations of GBCAs.

This case report has some limitations. We did not obtain the evidence of histochemically proven Gd deposition in the specific cerebral cortices. Therefore, we are not able to make a definitive statement. In addition, this patient had neurofibromatosis type 2 and underwent many surgical and radiotherapy treatments. Although these effects might have affected the MRI findings of the cerebral cortices, it is strongly suspected that Gd accumulation in the cortices caused the T_1_ shortening of the cerebral cortices.

In conclusion, we report a patient with T_1_ shortening in certain cerebral cortices after he had undergone more than 80 administrations of GBCAs. Based on our findings, we speculate that Gd deposition in the brain may be associated with the iron metabolism.

## References

[1] Kanda T Ishii K Kawaguchi H Kitajima K Takenaka D. High signal intensity in the dentate nucleus and globus pallidus on unenhanced T_1_-weighted MR images: relationship with increasing cumulative dose of a gadolinium-based contrast material. Radiology 2014; 270:834–841. 24475844 10.1148/radiol.13131669

[2] Errante Y Cirimele V Mallio CA Di Lazzaro V Zobel BB Quattrocchi CC. Progressive increase of T_1_ signal intensity of the dentate nucleus on unenhanced magnetic resonance images is associated with cumulative doses of intravenously administered gadodiamide in patients with normal renal function, suggesting dechelation. Invest Radiol 2014; 49:685–690. 24872007 10.1097/RLI.0000000000000072

[3] McDonald RJ McDonald JS Kallmes DF Intracranial gadolinium deposition after contrast-enhanced MR imaging. Radiology 2015; 275:772–782. 25742194 10.1148/radiol.15150025

[4] Kanda T Osawa M Oba H High signal intensity in dentate nucleus on unenhanced T_1_-weighted MR images: Association with linear versus macrocyclic gadolinium chelate administration. Radiology 2015; 275:803–809. 25633504 10.1148/radiol.14140364

[5] Kanda T Fukusato T Matsuda M Gadolinium-based contrast agent accumulates in the brain even in subjects without severe renal dysfunction: evaluation of autopsy brain specimens with inductively coupled plasma mass spectroscopy. Radiology 2015; 276:228–232. 25942417 10.1148/radiol.2015142690

[6] Stojanov D Aracki-Trenkic A Benedeto-Stojanov D. Gadolinium deposition within the dentate nucleus and globus pallidus after repeated administrations of gadolinium-based contrast agents-Current status. Neuroradiology 2016; 58:433–441. 26873830 10.1007/s00234-016-1658-1

[7] Kanda T Oba H Toyoda K Kitajima K Furui S. Brain gadolinium deposition after administration of gadolinium-based contrast agents. Jpn J Radiol 2016; 34:3–9. 26608061 10.1007/s11604-015-0503-5

[8] Thomsen HS Morcos SK Almén T ESUR Contrast Medium Safety Committee. Nephrogenic systemic fibrosis and gadolinium-based contrast media: updated ESUR contrast medium safety committee guidelines. Eur Radiol 2013; 23:307–318. 22865271 10.1007/s00330-012-2597-9

[9] Radbruch A Weberling LD Kieslich PJ Gadolinium retention in the dentate nucleus and globus pallidus is dependent on the class of contrast agent. Radiology 2015; 275:783–791. 25848905 10.1148/radiol.2015150337

[10] Radbruch A Weberling LD Kieslich PJ High-signal intensity in the dentate nucleus and globus pallidus on unenhanced T_1_-weighted images: Evaluation of the macrocyclic gadolinium-based contrast agent gadobutrol. Invest Radiol 2015; 50:805–810. 26523910 10.1097/RLI.0000000000000227

[11] Cao Y Huang DQ Shih G Prince MR. Signal change in the dentate nucleus on T_1_-weighted MR images after multiple administrations of gadopentetate dimeglumine versus gadobutrol. AJR Am J Roentgenol 2016; 206:414–419. 26700156 10.2214/AJR.15.15327

[12] Robic C Catoen S De Goltstein MC Idée JM Port M. The role of phosphate on Omniscan^®^ dechelation: an *in vitro* relaxivity study at pH 7. Biometals 2011; 24:759–768. 21390525 10.1007/s10534-011-9422-9

[13] Bressler JP Olivi L Cheong JH Kim Y Maerten A Bannon D. Metal transporters in intestine and brain: their involvement in metal-associated neurotoxicities. Hum Exp Toxicol 2007; 26:221–229. 17439925 10.1177/0960327107070573

[14] Ponka P. Heredity causes of disturbed iron homeostasis in the central nervous system. Ann NY Acad Sci 2004; 1012:267–281. 15105272 10.1196/annals.1306.022

[15] Ke Y Ming Qian Z. Iron misregulation in the brain: a primary cause of neurodegenerative disorders. Lancet Neurol 2003; 2:246–253. 12849213 10.1016/s1474-4422(03)00353-3

[16] Spatz H. Über den Eisennachweis im Gehirn, besonders in zentren des extrapyramidal-motorischen systems. Z Ges Neurol Psychiat 1922; 77:261–390.

[17] Hallgren B Sourander P. The effect of age on the non-haemin iron in the human brain. J Neurochem 1958; 3:41–51. 13611557 10.1111/j.1471-4159.1958.tb12607.x

[18] Aoki S Okada Y Nishimura K Normal deposition of brain iron in childhood and adolescence: MR imaging at 1.5T. Radiology 1989; 172:381–385. 2748819 10.1148/radiology.172.2.2748819

[19] Hirai T Korogi Y Sakamoto Y Hamatake S Ikushima I Takahashi M. T_2_ shortening in the motor cortex: effect of aging and cerebrovascular diseases. Radiology 1996; 199:799–803. 8638008 10.1148/radiology.199.3.8638008

[20] Korogi Y Hirai T Komohara Y T_2_ shortening in the visual cortex: effect of aging and cerebrovascular disease. AJNR Am J Neuroradiol 1997; 18:711–714. 9127035 PMC8338507

